# Supplementary material for: Notch/Her12 signalling modulates, motile/immotile cilia ratio downstream of Foxj1a in zebrafish left-right organizer
Source: eLife. 2017 Sep 6;6:e25165. doi: 10.7554/eLife.25165 (PMC5608511; doi:10.7554/eLife.25165)
Supplement: Supplementary file 2. [file elife-25165-supp2.pdf]

```
setOrigin <- function(xy, x0, y0, R, velFactor){
  x<- (xy[,4] - x0) / R
  y<- -(xy[,5] - y0) / R
  id<-xy[,2]
  omega <- atan2(x, y)
  r<- sqrt(x^2 + y^2)
  n<-length(x)
  a<-xy[,4][2:n] - xy[,4][1:(n-1)]
  b<-xy[,5][2:n] - xy[,5][1:(n-1)]
  alpha <-atan2(a, b)
  alpha.id <- which(alpha < 0)
  alpha[alpha.id] <- 2*pi + alpha[alpha.id]
  #speed <- sqrt(a^2 + b^2)
  #idx<-which(b == 0)
  #speed[idx] <- abs(a[idx])
  #idx2<-which(speed == 0)
  #a[idx2]
  #speed[idx2] <- abs(b[idx2])
  speed <- xy[,11]
  speed[is.na(speed)] <- 0
  p<- data.frame(x = x[1:(n-1)], y = y[1:(n-1)], id = id[1:(n-1)],
                 omega = omega[1:(n-1)], r = r[1:(n-1)],
                 alpha, speed = speed[2:(n)] * velFactor)
  return(p)
}


KVvectorField<-function(Data, Name, Scale){
  # do a magnitude/direction plot with radians
  drawKV(Name)
  mag<-Data$speed
  direction<-Data$alpha - pi/2
  xpos<-Data$x
  ypos<-Data$y
  vectorcol<-Data$id

vectorField(direction,mag,xpos,ypos,scale=Scale,vecspec="rad",col="b
lue",headspan=0.03)
}


KVnormalizedVectorField <- function(Data, Name, Scale1, Scale2)  {
  r.step = 0.15
  o.step = pi/36
  r.id = seq(0, 1.4 - r.step, r.step)
  o.id = seq(-pi, pi - o.step, o.step)
  data.avs<-NULL
  for (i in 1:length(r.id)){
    for(j in 1:length(o.id)){
      data.sub<-subset(Data, r > r.id[i]  & r < r.id[i] + r.step &
omega > o.id[j]  & omega < o.id[j] + o.step)
      speed.x<-(data.sub$speed)*cos(data.sub$alpha)
      speed.y<-(data.sub$speed)*sin(data.sub$alpha)
      vals<-data.frame(speed.x, speed.y, speed= data.sub$speed)
```

```
      if (length(data.sub[,1])>0){
        data.sub.m <- apply(data.sub,2, mean)
        #print(data.sub.m)
        #print(vals)
        vals.m <-apply(vals, 2, mean)
        speed.m<-sqrt(vals.m[1]^2 +  vals.m[2]^2)
        #print(vals.m)
        #print(speed.m)
        # print(data.avs)
        data.sub.m[["speed"]] <- speed.m
        #print(data.sub.m)
        alpha <-atan2(vals.m[2], vals.m[1])
        if(alpha < 0) alpha <- 2*pi + alpha
        data.sub.m[["alpha"]] = alpha
        #print(c("Alpha", alpha))
        #print(data.sub.m)
        data.avs <- rbind(data.avs, as.vector(data.sub.m))
      }
    }
  }
  colnames(data.avs)<-colnames(Data)
  data.avs<-data.frame(data.avs)
  par(mfrow = c(1,2))
  KVvectorField(Data, paste(Name, "(raw)"), Scale1)
  KVvectorField(data.avs, paste(Name, "(smoothed)"), Scale2)
  pdf(paste(Name,"_Flow.pdf", sep=""))
  par(mfrow = c(1,2))
  KVvectorField(Data, paste(Name, "(raw)"), Scale1)
  KVvectorField(data.avs, paste(Name, "(smoothed)"), Scale2)
  dev.off()
}

calcFlowHeatMaps<-function(data, Name){
  lim =2
  n<- 200
  L<- interp(data$x,data$y, data$speed,duplicate = "mean",
             xo=seq(-lim, lim, length=n), yo=seq(-lim, lim,
length=n))
  L.smooth<- image.smooth( na.exclude(L), theta = 0.040)
  for (i in 1:length(L$z[,1]))
    for (j in 1:length(L$z[1,])){
      if(is.na(L$z[i,j])) L.smooth$z[i,j] =NA
    }
  par(mfrow = c(1,2))
  drawKV(paste(Name, "Raw"))
  image.plot(L, main = "S", xlim = c(-1,1), ylim = c(-1,1),
zlim=c(0,30),add = T, horizontal =T, legend.lab = expression
(Flow~Velocity~(mu~m / s)))
  redrawKV()
  drawKV(paste(Name, "Smoothed"))
  image.plot(L.smooth, "S blured", xlim = c(-1,1), ylim =
c(-1,1),zlim=c(0,30), add = T, horizontal =T, legend.lab =
expression (Flow~Velocity~(mu~m / s)))
  redrawKV()
```

```
  pdf(paste(Name,"_FlowHeatMaps.pdf", sep=""))
  par(mfrow = c(1,2))
  drawKV(paste(Name, "Raw"))
  image.plot(L, main = "S", xlim = c(-1,1), ylim =
c(-1,1),zlim=c(0,30), add = T, horizontal =T, legend.lab =
expression (Flow~Velocity~(mu~m / s)))
  redrawKV()
  drawKV(paste(Name, "Smoothed"))
  image.plot(L.smooth, "S blured", xlim = c(-1,1), ylim = c(-1,1),
zlim=c(0,30),add = T, horizontal =T, legend.lab = expression
(Flow~Velocity~(mu~m / s)))
  redrawKV()
  dev.off()
}



drawKV <- function(nm){
  plot(c(-1.3, 1.3), c(-1.3,1.3), type = "n", asp=1,
     yaxt = 'n', xaxt='n', xlab ="", ylab = "",
       frame.plot = F, main = nm )
  #draw.circle(0,0, 1, lwd =1)
  #draw.circle(0,0, 0.5, lty = 1, lwd =1)
  text(-1.3, 0, "L")
  text(1.3, 0, "R")
  text(0, 1.3, "A")
  text(0, -1.3, "P")
}

redrawKV<- function(){
  #draw.circle(0,0, 1, lwd =1)
  #draw.circle(0,0, 0.5, lty = 1, lwd =1)
  text(-1.3, 0, "L")
  text(1.3, 0, "R")
  text(0, 1.3, "A")
  text(0, -1.3, "P")
  points(0,0, lwd =1)
  }


circularAnalysis1<-function(Coords, minR){
  idx<-which(Coords$r > minR)
  print(length(idx))
  H <-circular(Coords$omega,type="angle",units="radians", rotation =
"counter")
  plot(H[idx], rotation = "clock", zero =c(rad(90)), cex=1.03,
       stack=TRUE, add = T, axes = T, asp = 1, col = "darkgreen" )
  points(Coords$x[idx],Coords$y[idx], pch = 4,
         col = "darkgrey", cex = 0.5, symbol = "cross")
  draw.circle(0,0, minR, lty = 2, lwd =2)
  #Uniformity test
  rt<-rayleigh.test(H[idx], mu= NULL)
  p<-round(rt$p.value, digits = 3)
  for (i in seq(0, (3*pi)/2, by = pi/2)){
```

```
    rt<-rayleigh.test(H[idx], mu= circular(i))
    p<-c(p,round(rt$p.value, digits = 3))
  }
  l<-seq(0, (3*pi)/2, , by = pi/2)
  print(c(NA, l))
  print(p)
  text(0, 1.2, "A", cex = 1.2)
  text(0, 1.4, p[2], col = "darkgreen")
  mtext("R", 4, line = 0, las = 1)
  mtext(p[3], 4, line = 1, las = 1,  col = "darkgreen", cex = 0.7)
  text(0, -1.2, "P", cex = 1.2)
  text(0, -1.4, p[4], col = "darkgreen")
  mtext("L", 2, line = 0, las = 1)
  mtext(p[5], 2, line = 1, las = 1,  col = "darkgreen", cex = 0.7)
}

circularAnalysis2<-function(Coords, minR){
  idx<-which(Coords$r > minR)
  H <-circular(Coords$omega,type="angle",units="radians", rotation =
"counter")
#  drawKV(Title)
#  points(Coords$x[idx], Coords$y[idx], pch = 19, col = Coords$id,
cex = 0.5)
  rose.diag(-H[idx] + pi/2,
bins=24,shrink=0.5,xlim=c(-2,2),ylim=c(-2,2),
            axes=FALSE,prop=2)
  p<-NULL
  for (i in seq(0, (3*pi)/2, by = pi/2)){
    rt<-rayleigh.test(H[idx], mu= circular(i))
    p<-c(p,round(rt$p.value, digits = 3))
  }
  text(0, 1.2, "A", cex = 1.2)
  mtext("R", 4, line = 0, las = 1)
  text(0, -1.2, "P", cex = 1.2)
  mtext("L", 2, line = 0, las = 1)
}


analyzeAndPlot<-function(dat, Name){
  pdf(paste(Name,".pdf", sep=""))
  par(mfrow = c(1,3))
  drawKV(Name)
  points(dat$x,dat$y, pch = 19, col = dat$id, cex = 0.5)
  circularAnalysis1(dat, 0.5)
  circularAnalysis2(dat, 0.5)
  dev.off()
}
```
