# Supplementary material for: Notch/Her12 signalling modulates, motile/immotile cilia ratio downstream of Foxj1a in zebrafish left-right organizer
Source: eLife. 2017 Sep 6;6:e25165. doi: 10.7554/eLife.25165 (PMC5608511; doi:10.7554/eLife.25165)
Supplement: Transparent reporting form [file elife-25165-transrepform.pdf]

1st Floor
24 Hills Road
Cambridge CB2 1JP, UK

P 01223 855340
W elifesciences.org
T @elife

eLife's transparent reporting form

We encourage authors to provide detailed information *within their submission* to facilitate the interpretation and replication of experiments. If you have any questions, please contact us: editorial@elifesciences.org.

Sample-size estimation

- You should state whether an appropriate sample size was computed when the study was being designed
- You should state the statistical method of sample size computation and any required assumptions
- If no explicit power analysis was used, you should describe how you decided what sample (replicate) size (number) to use

Please outline where this information can be found within the submission (e.g., page numbers or figure legends), or explain why this information doesn't apply to your submission:

We did not compute an appropriated sample size when the study was being designed. The two main statistical methods used to calculate sample size in animal studies could not be correctly applied (**Guidelines for the design and statistical analysis of experiments using laboratory animals.** PMID: 12391400):

1. Power Analysis requires previously obtained data that is incompatible with exploratory research (e.g. effect size of interest, standard deviation).
2. Resource Equation Method, which is more adequate for exploratory assays, provided sample sizes we considered too small. This method considers as a rule of thumb that the degrees of freedom of the variable (E) should be approximately 10 to 20. Considering that "E" = "total number of animals" – "total number of test groups" (**How to Calculate Sample Size for Different Study Designs in Medical Research?** doi: 10.4103/0253-7176.116232), for example, in our live-imaging assay we have 9 test groups, so for E = 10, then "total number of animals" = 19 (2 animals per group), or for E = 20, then "total number of animals" = 29 (3 animals per group).

Replicates

- You should report how often each experiment was performed
- You should include a definition of biological versus technical replication
- The data obtained should be provided and sufficient information should be provided to indicate the number of independent biological and/or technical replicates
- If you encountered any outliers, you should describe how these were handled
- Criteria for exclusion/inclusion of data should be clearly stated

eLife Sciences Publications, Ltd is a limited liability non-profit non-stock corporation incorporated in the State of Delaware, USA, with company number 5030732, and is registered in the UK with company number FC030576 and branch number BR015634 at the address 1st Floor, 24 Hills Road, Cambridge CB2 1JP | August 2014

- High-throughput sequence data should be uploaded before submission, with a private link for reviewers provided (these are available from both GEO and ArrayExpress)

Please outline where this information can be found within the submission (e.g., page numbers or figure legends), or explain why this information doesn't apply to your submission:

The information on the number of replicates performed for each experiment (biological and/or technical, when applicable) is described in the "Materials and Methods" section of the manuscript.

We did not perform any outlier analysis, nor did we eliminate any data point on this criterion.

Data points were only eliminated if there was an obvious error associated with them (e.g. failure to produce a valid fluorescence curve in the qPCR) and if necessary or possible, the assay would be repeated in order to replace the data point. Zebrafish embryos that were deformed beyond what was reasonably expected were not used in the different assays (e.g. embryos that failed to reach bud stage were not used in the qPCR; embryos that at 7-somites stage did not present a normal development of the head were not used in the 2-photon live imaging assays).

Statistical reporting

- Statistical analysis methods should be described and justified
- Raw data should be presented in figures whenever informative to do so (typically when N per group is less than 10)
- For each experiment, you should identify the statistical tests used, exact values of N, definitions of center, methods of multiple test correction, and dispersion and precision measures (e.g., mean, median, SD, SEM, confidence intervals; and, for the major substantive results, a measure of effect size (e.g., Pearson's r, Cohen's d)
- Report exact p-values wherever possible alongside the summary statistics and 95% confidence intervals. These should be reported for all key questions and not only when the p-value is less than 0.05.

Please outline where this information can be found within the submission (e.g., page numbers or figure legends), or explain why this information doesn't apply to your submission:

The statistical methods used and their justification is described in the "Materials and Methods" section of the manuscript. The specific tests performed on each data set, the N per experimental group, Means ± SD, exact p values, etc. are further specified in the legends of the Figures or on the main text as needed.

Further information is available in the source data http://datadryad.org/review?doi=doi:10.5061/dryad.505q5.

eLife Sciences Publications, Ltd is a limited liability non-profit non-stock corporation incorporated in the State of Delaware, USA, with company number 5030732, and is registered in the UK with company number FC030576 and branch number BR015634 at the address 1st Floor, 24 Hills Road, Cambridge CB2 1JP | August 2014

(For large datasets, or papers with a very large number of statistical tests, you may upload a single table file with tests, Ns, etc., with reference to page numbers in the manuscript.)

Additional data files ("source data")

- We encourage you to upload relevant additional data files, such as numerical data that are represented as a graph in a figure, or as a summary table
- Where provided, these should be in the most useful format, and they can be uploaded as "Source data" files linked to a main figure or table
- Include model definition files including the full list of parameters used
- Include code used for data analysis (e.g., R, MatLab)
- Avoid stating that data files are "available upon request"

Please indicate the figures or tables for which source data files have been provided:

Figure 1, Figure 2, Figure 3, Figure 4, Figure 5, Figure 6, Figure S1, Figure S4, Figure S6 to S10 .

eLife Sciences Publications, Ltd is a limited liability non-profit non-stock corporation incorporated in the State of Delaware, USA, with company number 5030732, and is registered in the UK with company number FC030576 and branch number BR015634 at the address 1st Floor, 24 Hills Road, Cambridge CB2 1JP | August 2014
